# Anthropometry After Prematurity and Foetal Growth Restriction in Childhood and Adolescence

**DOI:** 10.1111/apa.70419

**Published:** 2025-12-24

**Authors:** Achim Fieß, Alica Hartmann, Stephanie D. Grabitz, Eva Mildenberger, Omar Hahad, Julia Winter, Alexander K. Schuster, Sandra Gißler, Dirk Wackernagel

**Affiliations:** ^1^ Department of Ophthalmology University Medical Center of the Johannes Gutenberg University Mainz Mainz Germany; ^2^ Division of Neonatology, Department of Pediatrics University Medical Center of the Johannes Gutenberg University Mainz Mainz Rhineland‐Palatinate Germany; ^3^ Department of Cardiology – Cardiology I, University Medical Center of the Johannes Gutenberg University Mainz Rhineland‐Palatinate Germany

**Keywords:** adolescence, anthropometry, childhood, foetal growth restriction, prematurity

## Abstract

**Aim:**

Limited evidence exists on how different degrees of prematurity and foetal growth deviations impact anthropometric outcomes during childhood and adolescence.

**Methods:**

The Gutenberg Prematurity Study Young (GPSY) is a retrospective cohort with prospective assessments of 4–17‐year‐olds born preterm or at term at the University Medical Center Mainz, Germany. Participants were classified by gestational age and birth weight into five categories: severely small for gestational age (SGA; < 3rd percentile), moderately SGA (3rd–< 10th percentile), appropriate for gestational age (AGA; 10th–90th percentile), moderately large for gestational age (LGA; > 90th–97th percentile) and severely LGA (> 97th percentile). Anthropometric parameters were analysed using multivariable regression models.

**Results:**

Among 949 participants (median age 12.0 years; 52% female), lower gestational age was associated with reduced height and smaller head circumference across all ages, and lower weight in early childhood and adolescence. SGA birth was consistently linked to shorter height, lower weight, lower BMI in school‐aged children, and smaller head circumference, especially at school age and adolescence. LGA birth was associated with increased height at school age and higher weight in early childhood and adolescence.

**Conclusion:**

These findings underscore the need for targeted growth monitoring and early interventions to optimise long‐term health trajectories.

## Summary

- Limited evidence exists on how different degrees of prematurity and deviations in foetal growth affect growth outcomes during childhood and adolescence.
- Children born preterm or small for gestational age showed reduced height, weight, BMI, and head circumference, whereas those born large for gestational age demonstrated increased growth parameters.
- These findings highlight the importance of targeted growth monitoring and early interventions to optimise long‐term health outcomes

**Abbreviations**
LGA: large for gestational age
SGA: small for gestational age

## 1 Introduction

Prematurity and foetal growth restriction have long‐term effects on health and development. These individuals often face delayed cognitive [1] and motor development [2], worse metabolic outcomes [3], cardiovascular disorders [4] and impaired renal health [5]. While the effects of prematurity and foetal growth restriction on anthropometric outcomes have been described individually, less is known about how different degrees of prematurity and foetal growth restriction, both independently and in combination, influence anthropometric development throughout childhood and adolescence.

The Danish National Birth Cohort Study analysed register‐based births, examining anthropometric data at 12 months, 5 years, 7 years, 11 years and 18 years of age. In childhood, a positive association between gestational age and body‐mass‐index (BMI) was observed, although this association disappeared by adolescence. Similarly, a positive association between gestational age and body height was evident in early childhood, with a weak difference persisting at the end of adolescence. Birth weight percentiles were not included in the analysis [6]. Other studies have found an association between prematurity and lower body weight [7] and lower head circumference [8] in childhood. A study of 135 preterm individuals born after 34 weeks of gestation found that those with foetal growth restriction were lighter and shorter compared to Dutch growth standards. The severity of foetal growth restriction was significantly associated with all measured growth outcomes [9].

The idea that prematurity and foetal growth restriction alter anthropometric outcomes is supported by the Developmental Origins of Health and Disease (DOHaD) theory, which suggests that genetic and environmental interactions during critical periods of embryonic, foetal, and neonatal development shape long‐term growth patterns [10].

This study aimed to explore how prematurity and foetal growth restriction alter anthropometric outcomes in children aged 4–17 years. It focused on understanding the effects of gestational age and different degrees of foetal growth restriction on anthropometry during childhood and adolescence. In addition, the study investigated how postnatal BMI normalisation, including its timing, contributes to differences in anthropometric development within this age group.

## 2 Materials and Methods

The Gutenberg Prematurity Study Young is a retrospective cohort study with prospective examinations and interviews conducted at the University Medical Center of the Johannes Gutenberg University Mainz (UMCM) in Germany. The study includes participants who were born preterm or at term between late 2003 and 2018 and were between 4 and 17 years of age at the time of enrolment. Each participant underwent one standardised in‐person examination at the study centre, during which anthropometric measurements were obtained. Every third randomly selected preterm infant with a gestational age of 33–36 weeks was invited to participate. In addition, all preterm newborns with a gestational age of 32 weeks or less were included. To form an age‐ and sex‐matched control group, eight term‐born individuals (four males and four females) with birth weights between the 10th and 90th percentiles were selected for each birth month from 2003 to 2018, as reported earlier [11, 12, 13, 14]. To examine the effects of varying levels of foetal growth independent of prematurity, the study also invited 60 full‐term individuals born SGA. These participants had birth weights below the tenth percentile. Additionally, 60 full‐term individuals were included who were born LGA, with birth weights above the ninetieth percentile. All participants were matched by age and sex.

Participants were stratified based on their gestational age. Group 1 included 380 individuals born at 37 weeks of gestation or later. Group 2 comprised 242 individuals born between 33 and 36 weeks and 6 days. Group 3 consisted of 180 individuals born between 29 and 32 weeks and 6 days. Group 4 included 147 individuals born before 29 weeks of gestation (see flow chart, Figure S1). The participants underwent examinations between 2022 and 2023. They also provided additional information about their medical history through surveys.

### 2.1 Assessment of Pre‐ and Postnatal History

Pre‐ and postnatal medical histories were obtained retrospectively from the participants' medical records. These historical data included gestational age in weeks, birth weight in kilograms, placental insufficiency, maternal smoking, preeclampsia, breastfeeding practices and perinatal complications. Placental insufficiency was defined as a documented diagnosis in the medical records, typically based on abnormal foetoplacental Doppler findings (increased umbilical artery pulsatility index, absent or reversed end‐diastolic flow) and/or clinical evidence of impaired placental function resulting in foetal growth restriction [15]. Breastfeeding was recorded as ‘yes’ if the mother reported any breastfeeding, regardless of duration or exclusivity. Moreover, birth weight percentiles were calculated based on the reference data by Voigt et al. [16].

### 2.2 Anthropometric Parameters and Postnatal BMI Normalisation

Anthropometric data were obtained from two sources. First, retrospective data were extracted from examination records of the standardised paediatric check‐up program (“U‐Untersuchungen”), as defined by the German Federal Joint Committee (G‐BA) [17] These included measurements from infancy, such as body height, weight, head circumference and BMI. Second, current anthropometric parameters were collected prospectively through direct clinical assessments conducted during the study visit. These included up‐to‐date measurements of body height, weight, head circumference and BMI. Percentiles were calculated using German reference standards. Body height was measured using a stadiometer with participants standing barefoot and body weight was measured using a calibrated digital scale. Head circumference was measured using a non‐stretchable measuring tape according to WHO guidelines. All measurements were performed by trained study personnel following standardised procedures.

At birth, the birth weight percentile adjusted for gestational age was calculated. BMI percentiles were calculated for subsequent timepoints to incorporate body length into the analysis of postnatal BMI normalisation using the KiGGS reference data [18] with the *childsds* package in R [19]. In the present study, we used BMI percentiles as a proxy for postnatal growth trajectories. Specifically, we examined postnatal BMI normalisation, defined as an increase of BMI above the 10th percentile for infants born SGA, or a decline into the 10th–90th percentile range for infants born LGA. BMI trajectories were visually explored up to 4 years of age to provide an overview of growth patterns. For the analytical definition of postnatal BMI normalisation, the 2‐year timepoint was chosen because most SGA infants complete postnatal BMI normalisation within the first 2 years of life, in line with previous research [20]. BMI was used as an indicator of postnatal normalisation due to its established role in paediatric growth monitoring, its clinical relevance in relation to long‐term metabolic outcomes and its widespread use in comparable studies [21, 22, 23].

### 2.3 Covariates

The analysis included the following covariates as potential influences on the main outcomes: weeks of prematurity measured as the number of weeks below the standard 40‐week term and abnormal foetal growth patterns defined as SGA and LGA.

### 2.4 Statistical Analysis

For descriptive analysis, absolute and relative frequencies were calculated for dichotomous variables, while means and standard deviations were determined for variables with a normal distribution. To compare anthropometric parameters across gestational age and birth weight percentile groups, analysis of variance was applied, with global *p*‐values calculated to identify significant differences in group means. Multivariable linear regression models were used to evaluate the effects of gestational age and abnormal foetal growth on anthropometric outcomes (height, weight, BMI and head circumference), with adjustments for age at examination and sex. This approach was chosen to account for age‐ and sex‐related differences in growth, allowing comparability of anthropometric outcomes across the predefined age groups. In a sensitivity analysis, maternal anthropometric data were also included as covariates. The relationship between postnatal BMI normalisation (yes/no) within 2 years and anthropometric outcomes was assessed using multivariable linear regression. In addition, we examined the rate of early BMI normalisation by calculating the change in BMI percentile from birth to 6 months of age, which was analysed as a continuous predictor in a separate linear model. Statistical analyses were conducted using R version 4.3.2., provided by the R Foundation for Statistical Computing in Vienna, Austria.

### 2.5 Ethics

All participants provided written informed consent prior to joining the study, adhering to the standards of Good Clinical Practice, Good Epidemiological Practice and the ethical guidelines of the Declaration of Helsinki. The study protocol and documentation were approved by the local ethics committee of the Medical Chamber of Rhineland‐Palatinate, Germany (reference no. 2021–15 830; original approval: May 5, 2021; most recent update: January 19, 2022).

## 3 Results

### 3.1 Participant Characteristics

The study included 949 individuals born preterm and at term, with a median age of 12.0 (IQR: 8.0, 15.0) years, of whom 495 were female (52%). Descriptive data and perinatal and postnatal parameters are presented in Table 1. Table 2 and Table 3 highlight significant anthropometric differences across gestational age groups and birth weight percentile groups. Anthropometric measurements in these tables refer to the values obtained at the study examination with one timepoint per participant. The smallest anthropometric measures were found in the gestational age of ≤ 28 weeks group and in the SGA groups.

**TABLE 1:** Characteristics of the study sample (*n* = 949).

	Group 1	Group 2	Group 3	Group 4
	GA ≥ 37 weeks	GA 33–36 weeks	GA 29–32 weeks	GA ≤ 28 weeks
Number of participants	380	242	180	147
Female participants	195 (51.3%)	120 (49.6%)	95 (52.8%)	85 (57.8%)
Age (y), at study examination	11.0 [8.0, 14.0]	12.0 [8.0, 14.8]	13.00 [8.8, 15.0]	11.0 [7.0, 15.0]
Neonatal parameters				
Birth weight (g), M ± SD	3385 ± 638	2335 ± 410	1558 ± 365	816 ± 250
Birth weight percentile, M ± SD	46.24 ± 33.40	33.98 ± 22.83	39.93 ± 22.85	37.63 ± 26.61
Severe SGA (BW percentile < 3)	30 (7.9%)	11 (4.5%)	5 (2.8%)	11 (7.5%)
Moderately SGA (BW percentile 3 to < 10)	30 (7.9%)	31 (12.8%)	13 (7.2%)	18 (12.2%)
AGA (BW percentile 10–90)	260 (68.4%)	197 (81.4%)	162 (90.0%)	116 (78.9%)
Moderately LGA (BW percentile > 90 to 97)	30 (7.9%)	2 (0.8%)	0 (0.0%)	2 (1.4%)
Severe LGA (BW percentile > 97)	30 (7.9%)	1 (0.4%)	0 (0.0%)	0 (0.0%)
Gestational age (weeks), M ± SD	38.98 ± 1.29	34.64 ± 1.04	30.86 ± 1.06	25.73 ± 1.65
Preeclampsia (yes)	7 (1.8%)	21 (8.7%)	33 (18.3%)	23 (15.6%)
Placental insufficiency (yes)	2 (0.5%)	8 (3.3%)	6 (3.3%)	11 (7.5%)
Maternal smoking (yes)	17 (4.5%)	7 (2.9%)	9 (5.0%)	15 (10.2%)
HELLP syndrome (yes)	0 (0.0%)	8 (3.3%)	21 (11.7%)	8 (5.4%)
Gestational diabetes (yes)	46 (12.1%)	19 (7.9%)	21 (11.7%)	7 (4.8%)
Breastfeeding (yes)	322 (84.7%)	183 (75.6%)	124 (68.9%)	78 (53.1%)

Abbreviations: AGA, appropriate for gestational age; BW, birth weight; GA, gestational age; HELLP, Haemolysis, Elevated Liver enzyme levels and Low Platelet levels; LGA, large for gestational age; M, Mean; SD, standard deviation; SGA, small for gestational age; y, year.

**TABLE 2:** Anthropometric data stratified by birth weight percentile groups.

Birth weight percentile groups	Severely SGA (< 3rd BW percentile)	Moderately SGA (3rd to < 10th BW percentile)	AGA (10th‐90th BW percentile)	Moderately LGA (> 90th to 97th BW percentile)	Severely LGA (> 97th BW percentile)	*p*‐value
Age group: early childhood (4–5 years)						
Number of participants	7	12	76	7	4	
Age (y), M ± SD	4.4 ± 0.5	4.6 ± 0.5	4.4 ± 0.5	4.7 ± 0.5	4.3 ± 0.5	0.45
Body height (cm), M ± SD	105.14 ± 7.45	105.91 ± 8.68	110.47 ± 8.92	117.29 ± 3.90	117.00 ± 5.83	0.02
Body weight (kg), M ± SD	16.55 ± 2.21	15.76 ± 2.94	18.42 ± 4.25	22.33 ± 4.33	22.55 ± 4.66	0.03
Body‐mass‐index, M ± SD	15.06 ± 1.28	13.97 ± 1.57	14.97 ± 1.81	16.16 ± 2.39	16.35 ± 2.05	0.003
Head circumference (cm), M ± SD	49.71 ± 2.66	49.65 ± 1.80	50.47 ± 2.03	51.64 ± 1.14	52.33 ± 4.59	0.11
Age group: school‐age (6–12 years)						
Number of participants	28	46	347	13	17	
Age (y), M ± SD	9.9 ± 1.7	9.0 ± 2.0	9.5 ± 2.0	9.8 ± 1.8	9.1 ± 2.1	0.25
Body height (cm), M ± SD	140.25 ± 13.14	136.88 ± 11.85	142.37 ± 14.28	148.46 ± 15.39	143.71 ± 14.26	0.05
Body weight (kg), M ± SD	32.93 ± 7.89	30.64 ± 7.45	35.68 ± 11.18	40.07 ± 11.10	36.02 ± 10.90	0.01
Body‐mass‐index, M ± SD	16.09 ± 2.10	16.22 ± 2.23	17.19 ± 3.05	17.82 ± 2.39	17.20 ± 3.04	0.09
Head circumference (cm), M ± SD	51.09 ± 2.24	52.37 ± 1.96	53.16 ± 2.14	54.19 ± 1.98	53.44 ± 3.24	< 0.001
Age group: adolescence (13–17 years)						
Number of participants	22	34	312	14	10	
Age (y), M ± SD	14.8 ± 1.3	15.3 ± 1.5	15.1 ± 1.5	14.4 ± 1.3	15.0 ± 1.3	0.38
Body height (cm), M ± SD	165.14 ± 11.51	163.21 ± 10.43	170.12 ± 9.31	171.75 ± 7.52	171.80 ± 8.70	0.001
Body weight (kg), M ± SD	56.35 ± 16.49	53.84 ± 10.58	61.32 ± 13.89	69.24 ± 16.73	76.88 ± 17.88	0.01
Body‐mass‐index, M ± SD	20.36 ± 4.58	20.19 ± 2.83	21.08 ± 3.86	23.21 ± 4.19	25.87 ± 6.32	0.06
Head circumference (cm), M ± SD	54.73 ± 1.90	54.94 ± 2.58	55.46 ± 2.48	56.36 ± 1.99	56.10 ± 2.20	< 0.001

Abbreviations: AGA, appropriate for gestational age; BW, birth; LGA, large for gestational age; M, Mean; SD, standard deviation; SGA, small for gestational age; y‐ year.

**TABLE 3:** Anthropometric data measured at study examination stratified by gestational age groups.

Gestational age groups	GA ≥ 37 GA weeks	33–36 GA weeks	29–32 GA weeks	≤ 28 GA weeks	*p*
Age group: early childhood (4–5 years)					
Number of participants	40	22	16	28	
Age (y), M ± SD	4.53 ± 0.51	4.55 ± 0.51	4.56 ± 0.51	4.21 ± 0.42	0.03
Body height (cm), M ± SD	113.15 ± 7.80	111.95 ± 7.78	111.03 ± 8.54	104.54 ± 9.06	0.001
Body weight (kg), M ± SD	19.84 ± 3.92	19.41 ± 5.08	18.48 ± 4.47	15.64 ± 2.65	0.08
Body‐mass‐index, M ± SD	15.41 ± 1.62	15.27 ± 1.98	14.76 ± 1.82	14.35 ± 1.98	0.13
Head circumference (cm), M ± SD	50.70 ± 2.47	51.00 ± 1.90	50.84 ± 1.67	49.55 (1.95)	0.06
Age group: school‐age (6–12 years)					
Number of participants	197	127	70	57	
Age (y), at study examination, M ± SD	9.57 ± 1.93	9.50 ± 2.05	9.21 ± 1.98	9.42 ± 2.10	0.63
Body height (cm), M ± SD	143.32 ± 13.80	142.11 ± 13.86	140.41 ± 13.57	138.36 ± 15.94	0.10
Body weight (kg), M ± SD	36.19 ± 10.97	35.23 ± 10.80	34.47 ± 10.14	32.14 ± 10.58	0.10
Body‐mass‐index, M ± SD	17.12 ± 2.84	16.99 ± 2.87	17.37 ± 2.94	16.48 ± 3.32	0.40
Head circumference (cm), M ± SD	53.31 ± 2.17	53.39 ± 2.00	52.59 ± 2.15	51.42 ± 2.45	< 0.001
Age group: adolescence (13–17 years)					
Number of participants	143	93	94	62	
Age (y), at study examination, M ± SD	14.88 ± 1.37	15.00 ± 1.39	15.04 ± 1.65	15.44 ± 1.50	0.10
Body height (cm), M ± SD	169.76 ± 9.05	171.53 ± 9.68	167.81 ± 10.10	167.39 ± 10.01	0.02
Body weight (kg), M ± SD	62.00 ± 14.99	63.37 ± 15.18	59.21 ± 13.56	58.28 ± 12.73	0.09
Body‐mass‐index, M ± SD	21.38 ± 4.36	21.37 ± 3.83	20.93 ± 3.91	20.67 ± 3.44	0.61
Head circumference (cm), M ± SD	55.63 ± 2.19	55.98 ± 2.74	55.20 ± 2.22	54.46 ± 2.58	0.001

Abbreviations: GA, gestational age; M, Mean; SD, standard deviation; y, year.

### 3.2 Uni‐ and Multivariable Analyses

Participants born at lower gestational age showed significantly reduced body height and smaller head circumference across all age groups, including early childhood, school age and adolescence. In addition, lower gestational age was associated with reduced body weight. This association was evident in the early childhood and adolescent group. No significant relationship was found between gestational age and BMI (Table 4). Being born SGA was associated with a shorter body height and lower body weight in all age groups. Being born SGA was associated with a lower BMI and a smaller head circumference in the school age group. In adolescence, it was further associated with a smaller head circumference. In contrast, individuals born LGA showed an increased body height during school age and higher body weight in early childhood and adolescence. Furthermore, individuals born LGA were associated with a higher BMI in adolescence (Table 4).

**TABLE 4:** Association analyses of the anthropometric parameters for children born preterm and term (*n* = 949).

Multivariable analysis adjusted for age and sex						
	Early childhood: 4–5 years (*n* = 106)		School‐aged children: 6–12 years (*n* = 451)		Adolescents: 13–17 years (*n* = 392)	
	Estimate (95% CI)	*p*	Estimate (95% CI)	*p*	Estimate (95% CI)	*p*
Body height [cm]						
Weeks of prematurity*	−0.42 (−0.66, −0.17)	0.001	−0.20 (−0.34, −0.05)	0.01	−0.28	0.002
SGA (< 10 percentile)	−5.99 (−9.28, −2.71)	< 0.001	−2.91 (−4.68, −1.15)	0.001	−6.72	< 0.001
LGA (> 90 percentile)	3.10 (−1.17, 7.38)	0.15	3.60 (0.92, 6.29)	0.01	0.51	0.77
Body weight [kg]						
Weeks of prematurity	−0.18 (−0.32, −0.05)	0.01	−0.15 (−0.30, 0.004)	0.06	−0.31 (−0.59, −0.03)	0.03
SGA (< 10 percentile)	−2.80 (−4.57, −1.03)	0.002	−3.52 (−5.45, −1.59)	0.003	−7.10 (−10.87, −3.33)	< 0.001
LGA (> 90 percentile)	2.46 (0.16, 4.76)	0.04	2.52 (−0.39, 5.42)	0.09	10.07 (4.42, 15.72)	< 0.001
Body‐mass‐index						
Weeks of prematurity	−0.04 (−0.12, 0.03)	0.21	−0.02 (−0.07, 0.04)	0.59	−0.04 (−0.13, 0.04)	0.31
SGA (< 10 percentile)	−0.63 (−1.59, 0.34)	0.20	−0.94 (−1.66, −0.22)	0.01	−0.90 (−2.03, 0.22)	0.11
LGA (> 90 percentile)	0.99 (−0.24, 2.21)	0.11	0.37 (−0.72, 1.45)	0.50	3.20 (1.52, 4.88)	< 0.001
Head circumference						
Weeks of prematurity	−0.08 (−0.16, −0.001)	0.05	−0.10 (−0.14, −0.06)	< 0.001	−0.09 (−0.14, −0.04)	< 0.001
SGA (< 10 percentile)	−0.96 (−2.01, 0.08)	0.07	−1.12 (−1.61, −0.63)	< 0.001	−0.75 (−1.42, −0.08)	0.03
LGA (> 90 percentile)	0.86 (−0.52, 2.24)	0.22	0.14 (−0.61, 0.88)	0.72	0.45 (−0.52, 1.43)	0.36

Abbreviations: Reference for SGA/LGA comparisons: AGA, appropriate for gestational age; LGA, large for gestational age; SGA, small for gestational age.

* Weeks of prematurity represents the number of weeks by which the gestation is shorter than the standard term pregnancy of 40 weeks.

In the sensitivity analysis with continuous parameters, a dose‐dependent association was observed across all age groups. Lower birth weight percentiles were linked to reduced body height, lower body weight and smaller head circumference (Table S1). When examining potential effect modification between gestational age and birth weight percentiles, a significant interaction was identified for BMI in adolescence (β_interaction = −0.003; 95% CI –0.006 to −0.001; *p* = 0.02) and for head circumference in school‐aged children (β_interaction = 0.003; 95% CI 0.002 to 0.004; *p* < 0.001). No interaction effects were observed for body height or body weight in any age group (Table S2).

A clear upward trend in anthropometric measurements was observed with increasing gestational age and birth weight percentile (Figure S2). Comprehensive data can be found in Figure S3.

The multivariable regression model was adjusted for the mother's height, weight and BMI (Table S3). The association between gestational age and body height remained significant. The association between gestational age and body weight remained evident only in the school‐age group. The previously identified relationships between birth weight percentiles and body height as well as body weight persisted for all age groups. Furthermore, an association between birth weight percentiles and BMI was observed in the school‐aged and adolescent group. Due to missing maternal data, the sample size was reduced to 662 participants.

### 3.3 Postnatal BMI Normalisation

Complete data up to 4 years for every timepoint (6 months, 1 year, 2 years, 4 years) were available for a subset of preterm participants at birth classified as moderately or severely SGA, accounting for 65 out of 89 cases (73%). Within this group, 51 out of the 65 cases (78%) showed BMI normalisation within the 4‐year period. Of the five preterm LGA cases, complete data for each timepoint up to 4 years of age were available for three individuals. All three individuals showed BMI normalisation.

Among term SGA infants, data up to 4 years were available for 43 out of 60 cases (72%) and 42 out of the 43 study participants (98%) showed BMI normalisation within 4 years. For term infants born moderately or severely LGA, data were available for 49 out of 60 cases, representing 82% of this cohort; 47 LGA cases out of 49 cases showed BMI normalisation after in the time frame of 4 years (96%).

**FIGURE 1:**
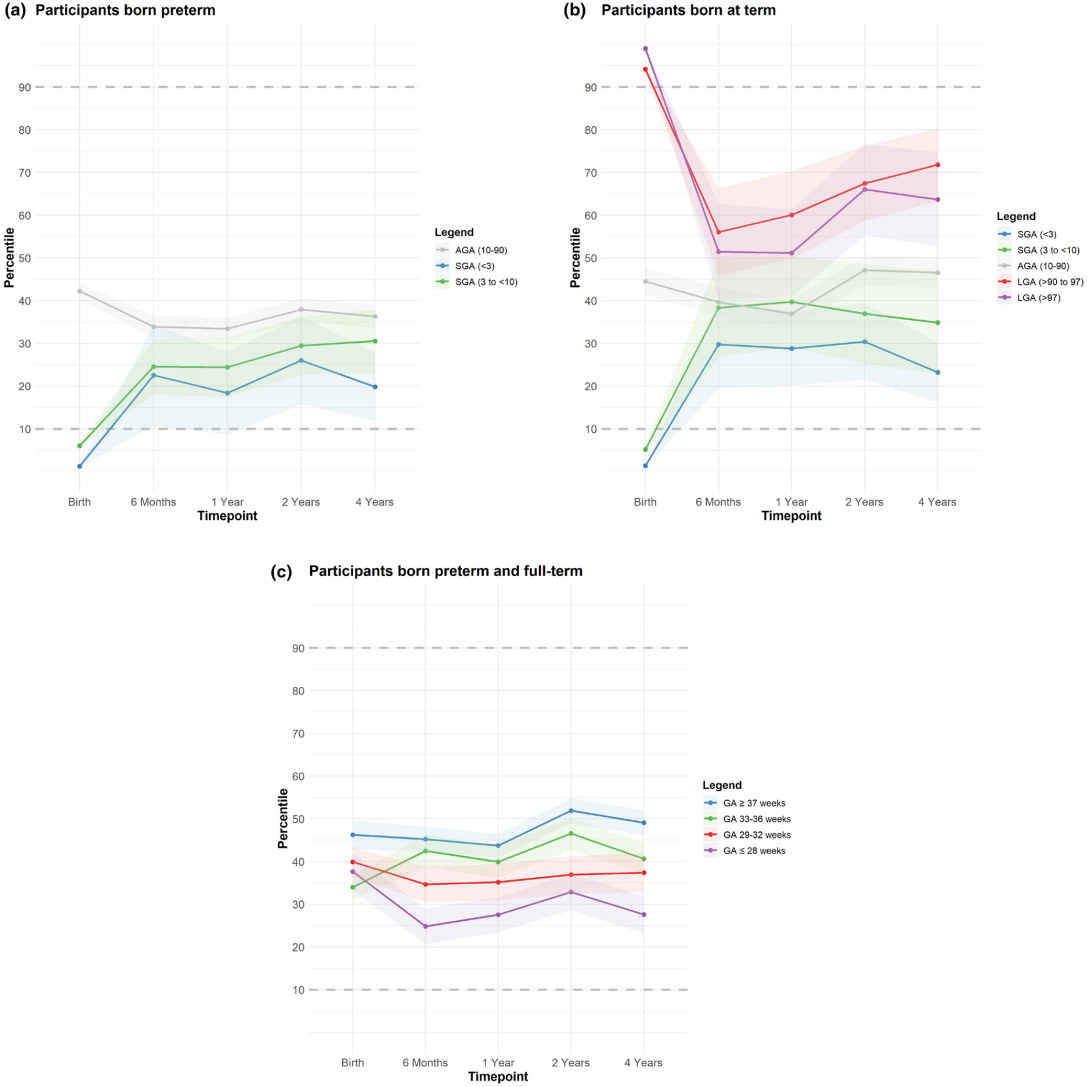
BMI percentile trajectories over 4 years in (A) preterm participants born small for gestational age (SGA) and appropriate for gestational age (AGA); (B) term participants born SGA, AGA, and large for gestational age (LGA); and (C) preterm and term participants across gestational age groups. (A) Preterm‐born participants by birth weight category: SGA < 3 (*n* = 19), SGA 3–< 10 (*n* = 46), AGA 10–90 (*n* = 370). (B) Term‐born participants by birth weight category: SGA < 3 (*n* = 21), SGA 3–< 10 (*n* = 22), AGA 10–90 (*n* = 220), LGA > 90–97 (*n* = 25), LGA > 97 (*n* = 25). (C) All participants by gestational age group: ≤ 28 weeks (*n* = 97), 29–32 weeks (*n* = 145), 33–36 weeks (*n* = 196), ≥ 37 weeks (*n* = 313). Shaded areas represent 95% confidence intervals. *n indicates the number of participants with complete BMI data available at all timepoints (birth, 6 months, 1 year, 2 years, and 4 years)*.

BMI percentile trajectories over the first 4 years are shown in Figure 1. Across birth weight and gestational age groups, varying patterns of BMI development were observed. SGA groups generally showed increases in BMI percentiles, while LGA groups tended to show decreases. AGA groups remained relatively stable. In the preterm groups, BMI trajectories appeared most variable in those born at ≤ 28 weeks of gestation based on visual assessment.

Participants born preterm and SGA with BMI normalisation in the first 2 years showed higher body weight, higher BMI and larger head circumference at the study examination (Figure S4a, Table S4). No such association was observed in participants born at term and SGA (Figure S4b, Table S5). No association was observed between the rate of BMI normalisation in the first 6 months and preterm or term birth (Tables S6 and S7).

## 4 Discussion

This study provided new insights into how gestational age, foetal growth and postnatal BMI normalisation influenced anthropometric outcomes in children aged 4–17 years, born both preterm and term. Unlike previous studies, this research uniquely examined the combined influence of gestational age and different levels of foetal growth. This approach offered a more comprehensive understanding of the impact of these characteristics on childhood and adolescent development.

Gestational age showed a dose‐dependent association with body height in all age groups. This suggests that factors altering growth during early development have lasting effects. In adolescence, each week of prematurity was associated with a 0.28 cm reduction in body height. Thus, a child born 10 weeks early was on average 2.8 cm shorter. This effect size, though modest on a per‐week basis, is clinically relevant when accumulated across several weeks and aligns with prior evidence on long‐term growth impairment in preterm populations.

Data from the Danish National Birth Cohort showed that lower gestational age was associated with lower body height at different timepoints up to 18 years of age. The effect of gestational age on body height decreased slightly with increasing age but remained significant across all age groups. That study did not distinguish between the effects of gestational age and birth weight percentile [6]. Another study examined 52 children aged 6–9 years, including 35 children born extremely preterm (< 28 weeks of gestational age) and 17 born at term. Children born preterm were significantly smaller in body size compared to term‐born children.

Moreover, our findings revealed an association between shorter height and SGA birth across all age groups. Adolescents born SGA were on average 6.72 cm shorter than non‐SGA peers. This substantial difference underscores the lasting impact of foetal growth restriction on longitudinal growth and highlights the importance of distinguishing between prematurity and growth‐related factors. One study described that children born SGA had a 7‐fold higher risk of short stature compared to the non‐SGA peers [24]. Another study on preterm infants showed an association between SGA births and reduced height [25]. Further studies showed associations between lower birth weight and smaller stature in childhood [26, 27, 28]. The consistent association between individuals born SGA and shorter height highlights the long‐term impact of foetal growth restriction on linear growth, beyond the effects of prematurity. Beyond physical growth, body height plays a role in psychosocial development. Recent studies have highlighted that height functions as an important social signal associated with peer perception, social integration and self‐esteem [29, 30, 31]. Individuals born SGA may therefore not only experience persistent height deficits but potentially also psychosocial consequences related to self‐perception and quality of life in later life. Although these outcomes were not assessed in the present study, they represent an important dimension of the long‐term impact of foetal growth restriction and should be addressed in future analyses.

In the present study, study participants in early childhood and adolescence showed a significant association between lower gestational age and lower childhood body weight. One study demonstrated that preterm birth (gestational age < 28 weeks) was linked to lower body weight in children aged 6–9 years compared to term‐born peers [32]. In another study, 497 preterm‐born and 95 term‐born children (≥ 37 weeks of gestation) were analysed at ages 8–12 years. The findings revealed that children born preterm had significantly lower body weight compared to their term‐born counterparts. Notably, birth weight percentiles were not considered in that analysis [7].

Furthermore, an association between being born SGA and lower body weight was found across all age groups. Another study observed that individuals born preterm and SGA had lower body weight persisting up to 12 years of age [33]. As the study participants were both preterm and SGA, it was not possible to distinguish between the effects of prematurity and foetal growth restriction. A clinical cohort examined 195 very low birth‐weight children (< 1500 g) born between 1977 and 1979 and followed them to ages of 8 and 20 years. At age 8, these children showed reduced weight compared to children born with a normal birth weight [34].

In our study individuals born LGA showed a higher weight in early childhood and adolescence. Other studies have demonstrated an association between higher birth weight and higher weight or obesity in childhood [35, 36, 37]. A Swedish study involving 195 936 women reported an increased risk of being overweight in adulthood among those born LGA [38]. This pattern may reflect an early predisposition towards increased adiposity among LGA individuals, reinforcing the importance of growth monitoring in this group.

After adjusting for maternal weight in our sensitivity analysis, birth weight percentile remained associated with lower body weight, while gestational age was only associated with body weight in early childhood. This suggests that foetal growth restriction plays a more distinct role than prematurity in influencing lower weight. Parental characteristics, however, appear to have a stronger effect on height. In contrast, body weight is more strongly shaped by lifestyle‐related factors [39]. We identified a relationship between maternal body weight and the body weight of participants, which underscores its inclusion as a confounding variable in our regression models.

Furthermore, our findings revealed specific associations between birth weight categories and BMI. Being born SGA was associated with a lower BMI in the school‐age group. In contrast, being born LGA was associated with a higher BMI in the adolescence group. The sensitivity analysis using continuous birth weight percentiles confirmed a dose‐dependent association between birth weight percentile and BMI across all age groups. In addition, we observed a small but statistically significant interaction between gestational age and birth weight percentile on BMI in adolescence. This suggests that the association between prematurity and BMI may vary across the birth weight spectrum. Although the effect size was modest, this finding indicates that birth weight percentile may slightly modify the influence of prematurity on later BMI.

Similar observations were reported in a study examining children born SGA and moderately preterm. That study showed that children born SGA, aged one to 5 years, had a lower BMI in early childhood [25]. Another study including children aged 7–10 years found an association between being born SGA and a lower BMI in childhood [40].

A Canadian perinatal registry, which included 4298 children, found that LGA born faced an increased risk of overweight at 10–11 years [41]. Another study with children aged 4–6 years showed an association between being born LGA and later overweight [42]. Furthermore, several studies described associations between birth weight and BMI in both childhood and adulthood [43, 44, 45].

In all age groups, there was an association between lower gestational age and smaller head circumference. A significant interaction between gestational age and birth weight percentile was observed in school‐aged children. This finding indicated that the negative association between prematurity and head circumference was more pronounced in those born with a lower birth weight percentile. However, this interaction was not observed in adolescence, suggesting that the modifying effect of birth weight percentile on head circumference may diminish over time. A study on newborns showed that babies born preterm, with a gestational age of 28–32 weeks, had smaller head circumferences at term. They remained smaller compared to babies born at term [46]. Another study found that preterm infants assessed at term had significantly smaller head circumferences than term‐born babies [47].

Additionally, we observed smaller head circumference values in the SGA group within the school‐aged cohort. In the analysis, using the birth weight percentile as a continuous parameter, this association was observed in all age groups. Another study examining 10‐year‐old children found that those born SGA had smaller head circumferences compared to AGA peers [48].

Moreover, our findings showed that BMI normalisation during the first 2 years of life was associated with higher body weight and BMI among individuals born preterm and SGA. In our cohort, BMI normalisation occurred mainly within the first 6 months of life. It is important to note that this analysis was based on a smaller subgroup, which limited the statistical power of the findings. A study of 403 individuals born very preterm showed that rapid weight gain during the first year of life was associated with higher BMI and fat percentage at 19 years of age [49]. Another study of children born SGA found that rapid BMI gain in the first 3 years of life was associated with a persistent risk of obesity at school age. Children who showed excessive catch‐up growth by the age of three had an increased risk of persistent overweight later in life [23]. This finding suggests that the timing and speed of postnatal BMI normalisation in preterm SGA infants may critically shape later weight and metabolic outcomes. These observations underline the clinical relevance of monitoring early postnatal growth patterns in SGA infants. The recent International Consensus Guideline on Small for Gestational Age recommends close growth surveillance during infancy and suggests that catch‐up growth should ideally occur before 3–4 years of age in order to support healthy long‐term development [50]. It should be noted that our analysis of timing of postnatal BMI normalisation was based on the change in BMI percentile from birth to 6 months and may therefore not fully capture different growth trajectories within this period. Future analyses using repeated‐measures or trajectory‐based modelling approaches may provide further insights into how distinct early growth patterns relate to later anthropometric outcomes.

The clinical relevance of our study lies in its detailed examination of the interplay between gestational age, foetal growth restriction and key anthropometric outcomes. These outcomes included body height, body weight, BMI and head circumference across different age groups. By using a stratified analysis of birth weight percentiles and gestational age categories, we provided a nuanced understanding of how these factors independently and interactively alter growth trajectories and long‐term health outcomes. Our findings showed that gestational age was strongly associated with childhood height and head circumference. In contrast, foetal growth restriction played a more important role in shaping BMI and body weight. Analyses of participants born LGA revealed contrasting risks, including higher BMI and weight. These results align with prior evidence linking higher birth weight to overweight and obesity. Together, these findings underline the necessity of early growth monitoring in these vulnerable groups.

## 5 Strengths and Limitations

This single‐centre, hospital‐based cohort study had certain limitations: A possible selection bias of predominantly white participants, which may have limited the generalisability of the findings. In addition, early anthropometric data were incomplete for some participants. Moreover, the study did not include standardised assessments of neurocognitive, metabolic, cardiovascular, renal or quality‐of‐life outcomes, which limited the ability to relate the observed anthropometric differences to functional long‐term health implications. Anthropometric percentiles were calculated using German reference standards, which are most appropriate for this population; however, the use of alternative international reference systems might have resulted in slight differences in percentile classification. Participants were born between 2003 and 2018, a period during which neonatal care improved substantially. Although age‐adjusted analyses reduce potential cohort effects, changes in neonatal care across birth years may have influenced early growth and cannot be fully excluded as a source of bias. Furthermore, the analysis involved multiple variables, which may have increased the risk of type I errors (false‐positive findings). Despite these limitations, the study stands out as one of the most detailed investigations into children and adolescents born preterm, encompassing a wide range of gestational ages and growth patterns. It benefits from thorough reviews of perinatal records, adherence to standardised measurement protocols and the implementation of investigator blinding to participants' birth‐related characteristics, ensuring methodological rigour.

## 6 Conclusion

This study showed that prematurity and foetal growth restriction had distinct effects on anthropometric outcomes, influencing growth trajectories from early childhood through adolescence. These findings highlight the need for early identification of at‐risk children and the implementation of targeted interventions to support healthy growth and reduce long‐term health risks.

## Author Contributions

**Achim Fieß:** conceptualization, validation, writing – original draft, writing – review and editing, formal analysis. **Alica Hartmann:** validation, writing – original draft, writing – review and editing, formal analysis. **Stephanie D. Grabitz:** validation, writing – review and editing. **Eva Mildenberger:** validation, writing – review and editing. **Omar Hahad:** validation, writing – review and editing. **Julia Winter:** validation, writing – review and editing. **Alexander K. Schuster:** conceptualization, validation, writing – review and editing. **Sandra Gißler:** validation, writing – review and editing, formal analysis. **Dirk Wackernagel:** validation, writing – review and editing, formal analysis.

## Funding

The Gutenberg Prematurity Study was supported by the Ernst und Berta‐Grimmke Stiftung, Stufe 1 support of the UM and the Else Kröner‐Fresenius‐Stiftung.

## Conflicts of Interest

Schuster AK receives research support from Allergan, Bayer, Heidelberg Engineering, PlusOptix and Norvartis. Fieß A, Hartmann A, Grabitz SD, Mildenberger E, Hahad O, Winter J, Gißler S, Wackernagel D: none.

## Supporting information


**Data S1:** Supporting Information.

## Data Availability

Data are available upon reasonable request. Interested researchers should make their requests to the coordinating PI of the Gutenberg Prematurity Study (Achim Fieß; achim.fiess@unimedizin-mainz.de).
