# Supplementary material for: Anthropometry After Prematurity and Foetal Growth Restriction in Childhood and Adolescence
Source: Acta Paediatr. 2025 Dec 24;115(4):902–12. doi: 10.1111/apa.70419 (PMC12975686; doi:10.1111/apa.70419)
Supplement: Supplementary file 1 — Data S1: Supporting Information. [file APA-115-902-s001.pdf]

Supplementary Tables

- Table S1. Association analyses of the anthropometric parameters (with continuous parameters) for children born preterm and term (n = 949)
- Table S2. Effect modification analyses of the anthropometric parameters (with continuous parameters) for children born preterm and term (n = 949)
- Table S3. Sensitivity analysis of the anthropometric parameters for children born preterm and term, adjusted for age, sex and maternal anthropometric parameters.
- Table S4. Postnatal BMI normalisation in individuals born small for gestational age in preterm (n=89).
- Table S5. Postnatal BMI normalisation in individuals born small for gestational age in term (n=60).
- Table S6. Percentile change over six months in individuals born small for gestational age and preterm (n=89).
- Table S7. Percentile change over six months in individuals born small for gestational age at term (n=60).

Supplementary Figures

- Figure S1. Study Design of the Gutenberg Prematurity Study Young (GPSY).
- Figure S2. 3D Surface Plots of a) body height [cm], b) body weight [kg], c) body mass index (BMI) [kg/m²], and d) head circumference [cm] across birth weight percentiles in preterm and term-born individuals.
- Figure S3. 3D Bar plots of a) body height, b) body weight, c) head circumference, and d) body mass index (BMI) [kg/m²]. Data are stratified by gestational age category and birth weight percentile category.
- Figure S4. A: Boxplots depicting body height (cm), body weight (kg), BMI (kg/m²), and head circumference (cm) in participants born preterm and classified as severely or moderately small for gestational age (SGA), stratified by the presence or absence of postnatal BMI normalisation. B: Boxplots illustrating the same measurements (body height, body weight, BMI, and head circumference) in participants born at term (gestational age ≥37 weeks) and classified as severely or moderately SGA, stratified by the presence or absence of postnatal BMI normalisation.

Figure S1. Study Design

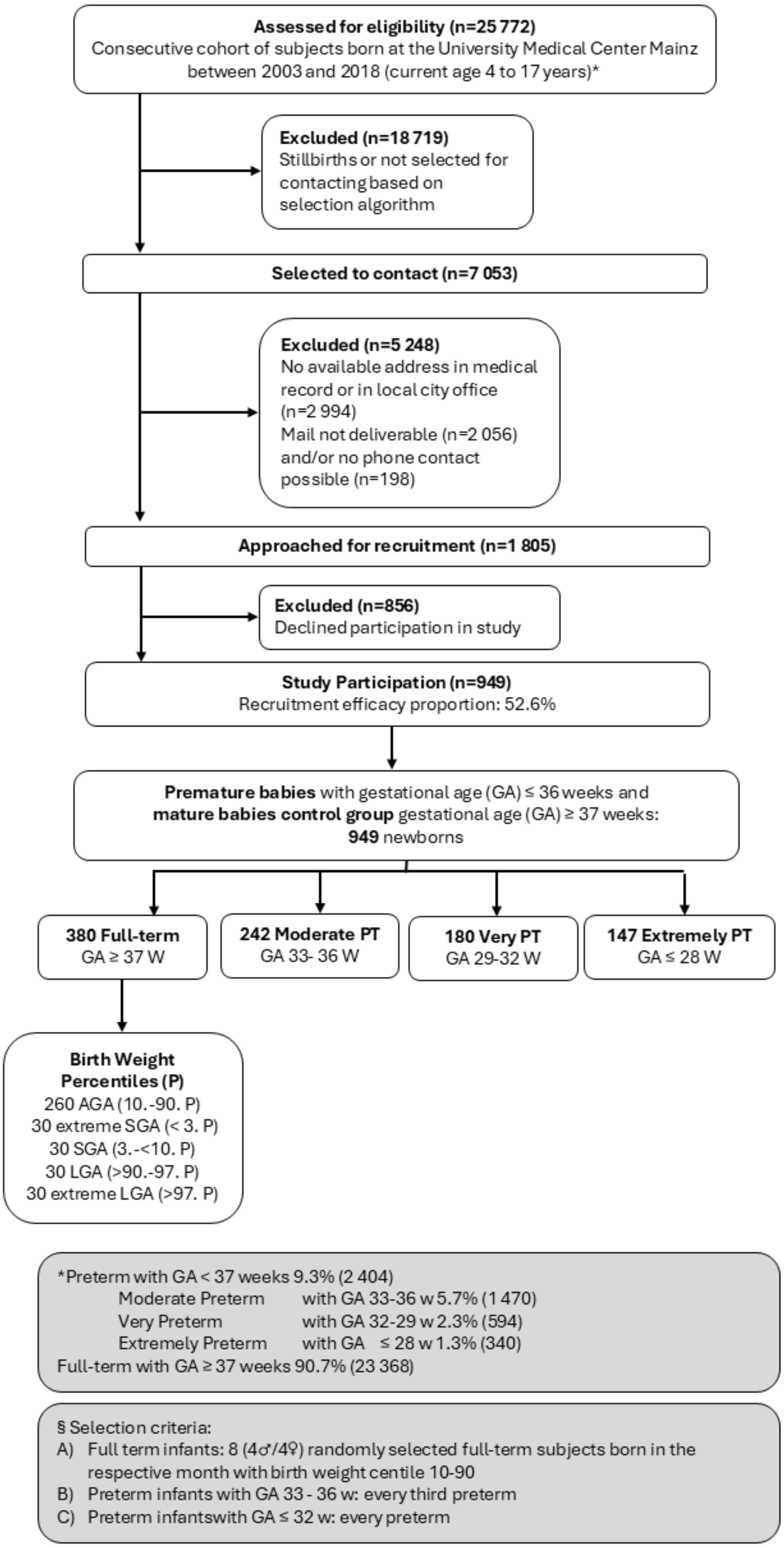

Figure S2. 3D Surface Plots of a) body height [cm], b) body weight [kg], c) body mass index (BMI) [kg/m²], and d) head circumference [cm] across birth weight percentiles in preterm and term-born individuals.

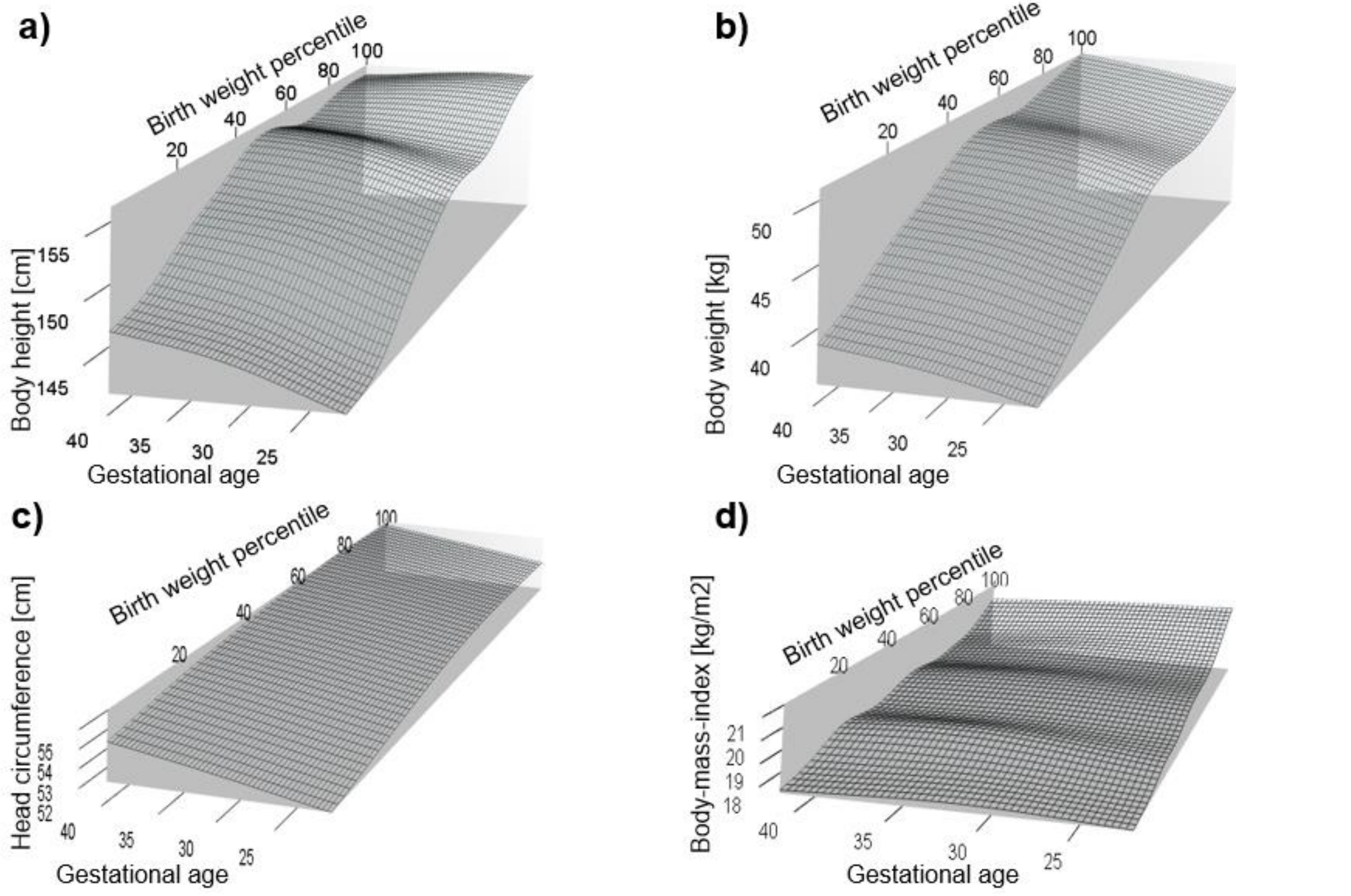

Figure S3. 3D Bar plots of a) body height, b) body weight, c) head circumference, and d) body mass index (BMI) [kg/m²] stratified by birth weight percentiles and gestational age

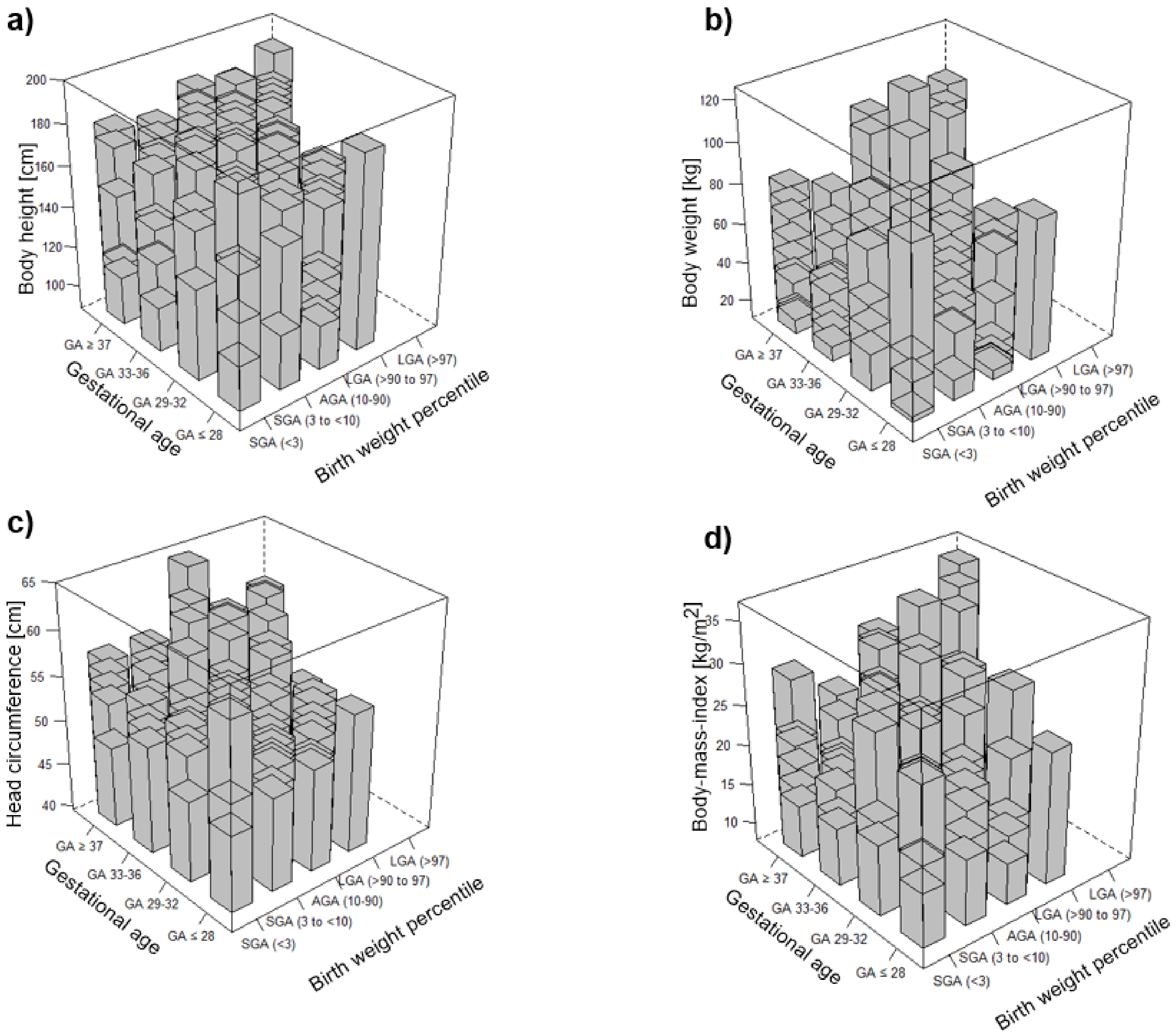

Figure S4. A: Boxplots depicting body height (cm), body weight (kg), BMI (kg/m²), and head circumference (cm) in participants born preterm and classified as severely or moderately small for gestational age (SGA), stratified by the presence or absence of postnatal BMI normalisation.

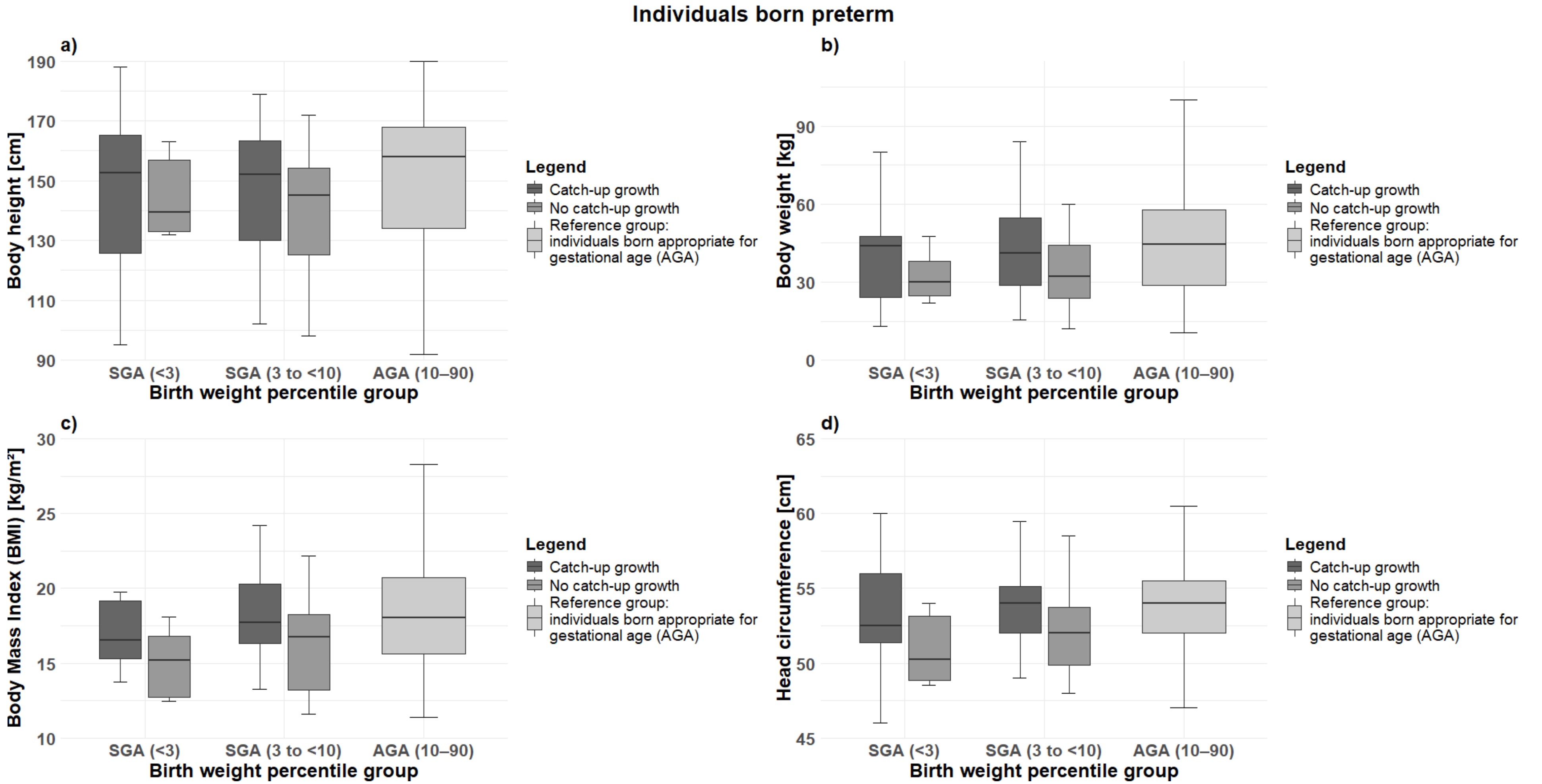

B: Boxplots illustrating the same measurements (body height, body weight, BMI, and head circumference) in participants born at term (gestational age ≥37 weeks) and classified as severely or moderately SGA, stratified by the presence or absence of postnatal BMI normalisation (catch-up growth).

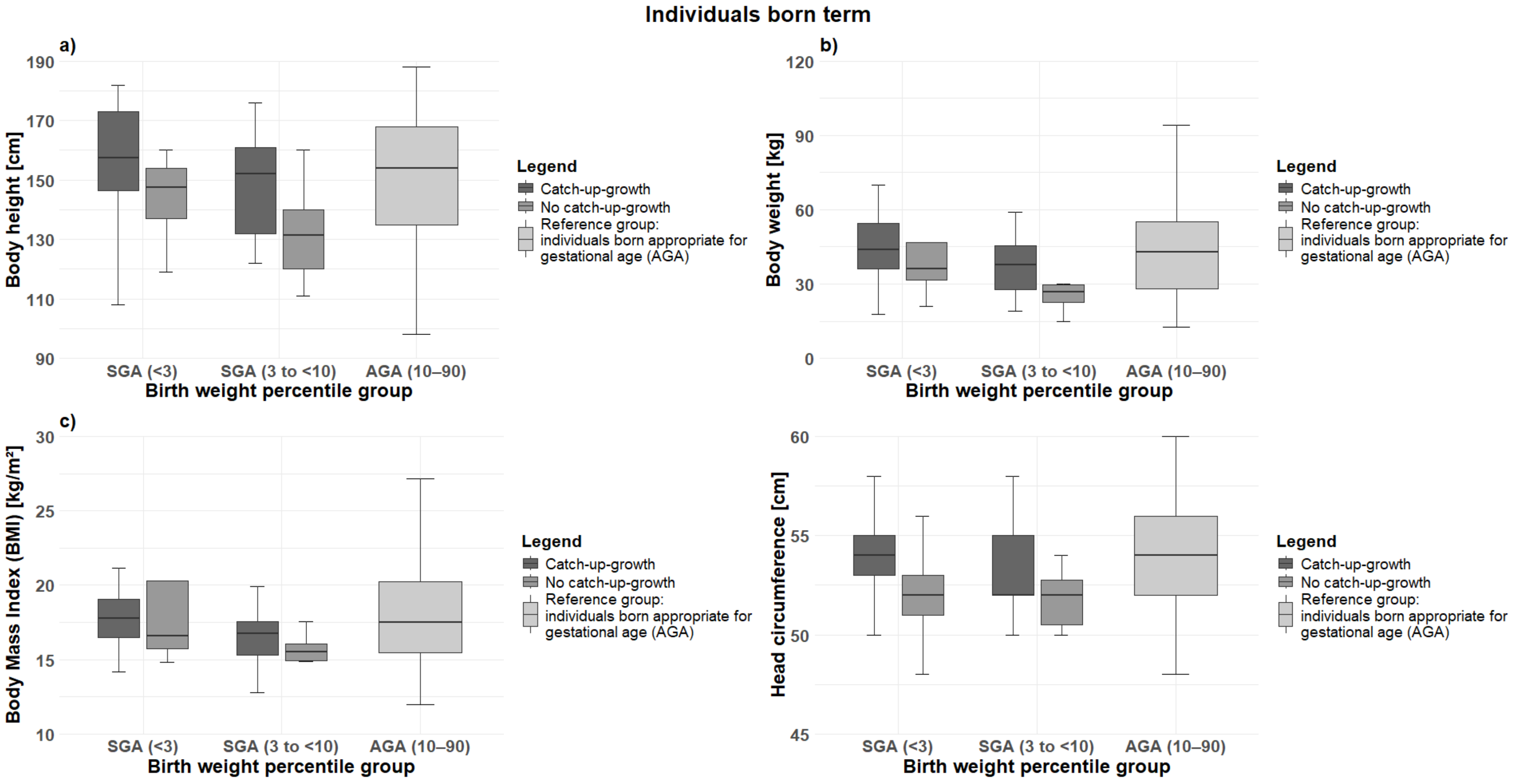

Table S1. Association analyses of the anthropometric parameters (with continuous parameters) for children born preterm and term (n = 949)

Multivariable analysis adjusted for age and sex						
	Early childhood group: 4 to 5 years		School-age group: 6 to 12 years		Adolescence: 13 to 17 years	
	Estimate (95% CI)	p-value	Estimate (95% CI)	p-value	Estimate (95% CI)	p-value
**Body height [cm]**						
Weeks of prematurity	-0.39 (-0.62, -0.17)	<0.001	-0.17 (-0.31, -0.03)	0.01	-0.26 (-0.42, -0.09)	0.003
Birth weight percentile	0.10 (0.06, 0.14)	<0.001	0.06 (0.04, 0.09)	<0.001	0.06 (0.03, 0.09)	<0.001
**Body weight [kg]**						
Weeks of prematurity	-0.19 (-0.32, -0.07)	0.003	-0.11 (-0.26, 0.04)	0.16	-0.37 (-0.64, -0.09)	0.01
Birth weight percentile	0.04 (0.02, 0.07)	<0.001	0.07 (0.05, 0.10)	<0.001	0.12 (0.08, 0.17)	<0.001
**Body-mass-index**						
Weeks of prematurity	-0.06 (-0.12, 0.01)	0.11	-0.001 (-0.06, 0.06)	0.99	-0.07 (-0.15, 0.01)	0.11
Birth weight percentile	0.01 (-0.002, 0.02)	0.11	0.02 (0.01, 0.03)	<0.001	0.03 (0.01, 0.04)	<0.001
**Head circumference**						
Weeks of prematurity	-0.08 (-0.16, -0.01)	0.03	-0.09 (-0.12, -0.05)	<0.001	-0.09 (-0.14, -0.04)	<0.001
Birth weight percentile	0.02 (0.003, 0.03)	0.02	0.02 (0.01, 0.02)	<0.001	0.01 (0.01, 0.02)	<0.001

* Weeks of prematurity represents the number of weeks by which the gestation is shorter than the standard term pregnancy of 40 weeks. SGA - small for gestational age; LGA - large for gestational age. Reference for SGA/LGA comparisons: AGA - appropriate for gestational age.

Table S2. Effect modification analyses of the anthropometric parameters (with continuous parameters) for children born preterm and term (n = 949)

**Multivariable analysis adjusted for age and sex**						
	Early childhood group: 4 to 5 years		School-age group: 6 to 12 years		Adolescence: 13 to 17 years	
	Estimate (95% CI)	p-value	Estimate (95% CI)	p-value	Estimate (95% CI)	p-value
**Body height [cm]**						
Weeks of prematurity	-0.36 (-0.76, 0.04)	0.07	-0.21 (-0.44, 0.02)	0.07	-0.47 (-0.79, -0.16)	0.003
Birth weight percentile	0.09 (0.03, 0.14)	0.002	0.06 (0.03, 0.10)	<0.001	0.04 (0.001, 0.08)	0.04
Weeks of prematurity* Birth weight percentile	-0.001 (-0.009, 0.007)	0.78	0.002 (-0.004, 0.01)	0.51	0.005 (-0.0005, 0.01)	0.07
**Body weight [kg]**						
Weeks of prematurity	-0.17 (-0.38, 0.05)	0.12	-0.23 (-0.47, 0.01)	0.06	-0.02 (-0.53, 0.48)	0.93
Birth weight percentile	0.05 (0.02, 0.08)	0.002	0.06 (0.03, 0.09)	<0.001	0.16 (0.10, 0.23)	<0.001
Weeks of prematurity* Birth weight percentile	-0.001 (-0.005, 0.004)	0.78	0.004 (-0.002, 0.01)	0.19	-0.007 (-0.02, 0.002)	0.11
**Body-mass-index**						
Weeks of prematurity	-0.01 (-0.13, 0.10)	0.81	-0.06 (-0.15, 0.03)	0.17	0.08 (-0.07, 0.23)	0.28
Birth weight percentile	0.01 (-0.002, 0.03)	0.08	0.01 (0.001, 0.03)	0.03	0.04 (0.02, 0.06)	<0.001

Weeks of prematurity* Birth weight percentile	-0.001 (-0.003, 0.001)	0.40	0.002 (-0.0002, 0.003)	0.09	-0.003 (-0.006, -0.001)	0.02
**Head circumference**						
Weeks of prematurity	-0.14 (-0.26, -0.01)	0.04	-0.19 (-0.26, -0.13)	<0.001	-0.08 (-0.17, 0.004)	0.06
Birth weight percentile	0.01 (-0.01, 0.03)	0.27	0.007 (-0.002, 0.01)	0.11	0.02 (0.004, 0.03)	0.01
Weeks of prematurity* Birth weight percentile	0.001 (-0.001, 0.004)	0.31	0.003 (0.002, 0.004)	<0.001	-0.0001 (-0.002, 0.002)	0.90

Table S3. Sensitivity analysis of the anthropometric parameters for children born preterm and term, adjusted for age, sex and maternal anthropometric parameters.

Multivariable analysis						
	Early childhood group: 4 to 5 years		School-age group: 6 to 12 years		Adolescence: 13 to 17 years	
	Estimate (95% CI)	p-value	Estimate (95% CI)	p-value	Estimate (95% CI)	p-value
**Body height [cm]**						
Weeks of prematurity	-0.30 (-0.61, 0.01)	0.06	-0.19 (-0.36, -0.02)	0.03	-0.25 (-0.45, -0.05)	0.02
Birth weight percentile	0.10 (0.05, 0.16)	<0.001	0.05 (0.02, 0.07)	<0.001	0.05 (0.01, 0.08)	0.01
**Body weight [kg]**						
Weeks of prematurity	-0.20 (-0.37, -0.03)	0.02	-0.13 (-0.31, 0.04)	0.14	-0.29 (-0.61, 0.03)	0.08
Birth weight percentile	0.05 (0.02, 0.08)	0.002	0.06 (0.04, 0.09)	<0.001	0.09 (0.04, 0.15)	0.001
**Body-mass-index**						
Weeks of prematurity	-0.08 (-0.16, 0.01)	0.08	0.01	0.84	-0.06 (-0.15, 0.04)	0.23
Birth weight percentile	0.01 (-0.01, 0.02)	0.36	0.02	0.003	0.02 (0.004, 0.04)	0.02

* Weeks of prematurity represents the number of weeks by which the gestation is shorter than the standard term pregnancy of 40 weeks. SGA - small for gestational age; LGA - large for gestational age. Reference for SGA/LGA comparisons: AGA - appropriate for gestational age.

Table S4. Postnatal BMI normalisation in individuals born small for gestational age in preterm (n=89).

	Multivariable analysis, adjusted for age*	
	Estimate (95% CI)	p-value
**Body height [cm]**		
Postnatal BMI normalisation after two years (yes)	3.88 (-0.25, 8.00)	0.07
Weeks of prematurity	-0.09 (-0.54, 0.35)	0.68
Birth weight percentile	-0.25 (-0.98, 0.48)	0.49
**Body weight [kg]**		
Postnatal BMI normalisation after two years (yes)	8.45 (3.90, 12.99)	<0.001
Weeks of prematurity	0.05 (-0.44, 0.55)	0.83
Birth weight percentile	-0.23 (-1.03, 0.57)	0.57
**Body-mass-index**		
Postnatal BMI normalisation after two years (yes)	2.68 (1.22, 4.14)	<0.001
Weeks of prematurity	0.01 (-0.15, 0.17)	0.87
Birth weight percentile	0.03 (-0.23, 0.28)	0.84
**Head circumference**		
Postnatal BMI normalisation after two years (yes)	1.02 (0.001, 2.03)	0.05
Weeks of prematurity	-0.17 (-0.28, -0.06)	0.003
Birth weight percentile	0.12 (-0.06, 0.30)	0.18

* Weeks of prematurity deficit represents the number of weeks by which the gestation is shorter than the standard term pregnancy of 40 weeks. Results were adjusted for age but not for sex due to the small subgroup analysis being limited to male participants.

Table S5. Postnatal BMI normalisation in individuals born small for gestational age in term (n=60).

	Multivariable analysis, adjusted for age and sex	
	Estimate (95% CI)	p-value
**Body height [cm]**		
Postnatal BMI normalisation after two years (yes)	2.03 (-3.19, 7.25)	0.44
Weeks of prematurity*	1.56 (-0.03, 3.14)	0.05
Birth weight percentile	0.28 (-0.67, 1.23)	0.56
**Body weight [kg]**		
Postnatal BMI normalisation after two years (yes)	-0.63 (-7.33, 6.07)	0.85
Weeks of prematurity	1.29 (-0.75, 3.33)	0.21
Birth weight percentile	-0.51 (-1.75, 0.73)	0.41
**Body-mass-index**		
Postnatal BMI normalisation after two years (yes)	-0.78 (-2.98, 1.42)	0.48
Weeks of prematurity	0.23 (-0.44, 0.89)	0.49
Birth weight percentile	-0.23 (-0.63, 0.17)	0.25
**Head circumference**		
Postnatal BMI normalisation after two years (yes)	0.57 (-0.89, 2.02)	0.43
Weeks of prematurity	-0.10 (-0.54, 0.35)	0.67
Birth weight percentile	0.16 (-0.11, 0.43)	0.25

* Weeks of prematurity represents the number of weeks by which the gestation is shorter than the standard term pregnancy of 40 weeks.

Table S6. Percentile change over six months in individuals born small for gestational age and preterm (n=89).

	Multivariable analysis, adjusted for age*	
	Estimate (95% CI)	p-value
**Body height [cm]**		
Percentile change up to six months	0.002 (-0.08, 0.08)	0.95
Weeks of prematurity*	-0.38 (-0.89, 0.13)	0.14
Birth weight percentile	-0.09 (-0.86, 0.68)	0.82
**Body weight [kg]**		
Percentile change up to six months	0.01 (-0.08, 0.10)	0.81
Weeks of prematurity	-0.13 (-0.73, 0.46)	0.65
Birth weight percentile	0.23 (-0.65, 1.12)	0.60
**Body-mass-index**		
Percentile change up to six months	0.006 (-0.03, 0.04)	0.70
Weeks of prematurity	-0.01 (-0.23, 0.20)	0.91
Birth weight percentile	0.13 (-0.19, 0.45)	0.43
**Head circumference**		
Percentile change up to six months	0.01 (-0.08, 0.10)	0.81
Weeks of prematurity	-0.13 (-0.73, 0.46)	0.65
Birth weight percentile	0.23 (-0.65, 1.12)	0.60

* Weeks of prematurity represents the number of weeks by which the gestation is shorter than the standard term pregnancy of 40 weeks. Results were adjusted for age but not for sex due to the small subgroup analysis being limited to male participants.

Table S7. Percentile change over six months in individuals born small for gestational age at term (n=60).

	Multivariable analysis, adjusted for age and sex	
	Estimate (95% CI)	p-value
**Body height [cm]**		
Percentile change up to six months	0.02 (-0.05, 0.10)	0.55
Weeks of prematurity	1.43 (-0.32, 3.18)	0.11
Birth weight percentile	0.14 (-0.92, 1.20)	0.79
**Body weight [kg]**		
Percentile change up to six months	0.03 (-0.05, 0.12)	0.44
Weeks of prematurity	1.79 (-0.18, 3.77)	0.07
Birth weight percentile	-0.43 (-1.62, 0.77)	0.48
**Body-mass-index**		
Percentile change up to six months	0.01 (-0.02, 0.03)	0.63
Weeks of prematurity	0.36 (-0.20, 0.93)	0.20
Birth weight percentile	-0.12 (-0.46, 0.22)	0.48
**Head circumference**		
Percentile change up to six months	0.01 (-0.02, 0.03)	0.61
Weeks of prematurity	-0.24 (-0.69, 0.20)	0.27
Birth weight percentile	0.12 (-0.15, 0.39)	0.37

* Weeks of prematurity represents the number of weeks by which the gestation is shorter than the standard term pregnancy of 40 weeks.
